# Individual differences and diversity in human physiological responses to light

**DOI:** 10.1016/j.ebiom.2021.103640

**Published:** 2022-01-10

**Authors:** Manuel Spitschan, Nayantara Santhi

**Affiliations:** a Max Planck Institute for Biological Cybernetics, Tübingen, Germany; b Department of Sport and Health Sciences, Technical University of Munich, Munich, Germany; c Department of Experimental Psychology, University of Oxford, United Kingdom; d Department of Psychology, Northumbria University, United Kingdom

**Keywords:** Non-visual effects of light, Circadian rhythms, Individual differences, Diversity, Data sharing, Open science

## Abstract

Exposure to light affects our physiology and behaviour through a pathway connecting the retina to the circadian pacemaker in the hypothalamus – the suprachiasmatic nucleus (SCN). Recent research has identified significant individual differences in the non-visual effects of light,mediated by this pathway. Here, we discuss the fundamentals and individual differences in the non-visual effects of light. We propose a set of actions to improve our evidence database to be more diverse: understanding systematic bias in the evidence base, dedicated efforts to recruit more diverse participants, routine deposition and sharing of data, and development of data standards and reporting guidelines.

## 1 Introduction

The physiology and behaviour of all living species are rhythmic and synchronized to the 24-hour solar day. In humans, these daily rhythms are synchronized by a central pacemaker – the circadian clock – located in the suprachiasmatic nucleus (SCN) in the hypothalamus [1]. The SCN receives information about environmental illumination through the retinohypothalamic tract (RHT), enabling it to synchronize our internal time to the external light-dark cycle. This clock is the origin of many circadian rhythms in physiology, including the secretion of hormones, variation in body temperature, and performance, which persist even in the absence of a *zeitgeber*, a stimulus synchronizing internal with external time. Besides influencing the clock in this fundamental way through circadian entrainment, light also directly influences melatonin production, alertness, cognition, and other functions [2]. The term “non-visual effects of light” is often used as an umbrella term for these effects.

The non-visual effects of light are mediated by a multi-component photoreceptive system, consisting of rods, cones and the intrinsically photosensitive retinal ganglion cells (ipRGCs). The ipRGCs have unique wavelength-dependent sensitivity arising from the expression of the photopigment melanopsin. Melanopsin is sensitive to short-wavelength light [3], [4], [5], [6], with a peak sensitivity around 480 nm, and encodes environmental light levels independent of the canonical photoreceptors — the cones and the rods, which allow us to see the colourful, detailed and moving world around us. Historically, its discovery in the mammalian eye in the late 1990s [7,8] coincided with several converging lines of evidence pointing to an additional photoreceptor system mediating light-induced melatonin suppression during the biological night [9], [10], [11].

The seminal discovery of light-induced suppression of endogenous melatonin led to a focused effort, over the past 40 years, to uncover how light regulates neuroendocrine, circadian and other non-visual processes. Critically, light exposure at the wrong time, such as at night, causes our circadian rhythms to desynchronize from the sleep wake-cycle [12]. The term ‘circadian misalignment’ is frequently used to describe this situation, which occurs during shift-work, transmeridian travel and extended work shifts [13]. Prolonged light exposure, especially after dusk, can also adversely impact sleep: [1] The artificial light we expose ourselves to in the evening at home suppresses sleepiness and delays sleep onset [14], [15], [16]. Given this important role of light exposure, a critical translational goal is to maximize its beneficial effects and minimize its possible adverse effects.

## 2 Individual differences in the non-visual effects of light

The non-visual effects of light depend on timing [95], intensity, [96] wavelength [[97], [98]] and duration [99] of light exposure, and prior light history [2]. There is converging evidence for individual differences in the non-visual response to light in healthy participants, which are not easy to explain away [17], [18], [19], [20], [21], [22]. A recent study by Philipps et al [17]. demonstrated that melatonin suppression by evening light could be subject to substantial individual differences. The most sensitive observer was almost 60 times more sensitive than the least sensitive observer in their data set. Specifically, the most sensitive observer experienced 50% melatonin suppression at ∼10 lx (approximately similar to dim reading light illumination). In contrast, for the least sensitive observer, 50% melatonin suppression occurred at 400 lx (similar to bright home illumination). Preceding light exposure could not account for these differences, also termed "photic history", desensitizing the evening response to light [23], [24], [25], [26], [27], [28].

What gives rise to the stark individual differences in sensitivity to light (recently reviewed by Chellappa [29])? Retinal illuminance may vary between individuals due to progressive age-dependent changes in the lens, causing a reduction in the amount of short-wavelength light passing through the eye and scatter [30], [31], [32], [33], and differences in pupil size [22,32,34]. Health status and the use of medications may also change non-visual sensitivity to light [35]. Furthermore, there is evidence for genetic variability changing the sensitivity of melanopsin signalling characteristics [36,37].) Long-term ‘photic history’ at various time scales, including seasonal changes in the light environment [38], [39], [40], [41], [42] also contribute to individual differences in sensitivity to non-visual effects of light [43,44]. A complete understanding of all the factors underlying the observed individual differences eludes us, necessitating more principled research.

## 3 Standardization and the prospect of a non-visual standard observer

A significant accomplishment in driving forward the integration of physiologically relevant knowledge in this domain was the development of the international standard CIE S 026/E:2018 by the International Commission on Illumination (CIE) [45], based on prior scientific consensus [6]. CIE S026/E:2018 standardizes the spectral weighting functions for cones, rods and melanopsin, thereby standardizing the metrology for characterizing light concerning its non-visual effects on humans [46], and paving the way for building and testing mechanistic models of circadian and neuroendocrine phototransduction.

The concept of the “standard observer” is pervasive in light, vision, and colour science. One such “standard observer” is implicit in the photopic luminosity curve V(λ) underlying photometric measurements of light [47]. The photopic luminosity curve describes the spectral sensitivity of human observers in response to flickering light in a paradigm called *heterochromatic flicker photometry*, which primarily reflects contributions from the L and M cones [48]. Importantly, different psychophysical paradigms may yield different V(λ)-like curves [49]. It is well known that V(λ) underestimates sensitivity to short-wavelength light even in average data [48], and indeed, “no one is the mean” [50]. V(λ) is implicit in the *candela*, the only SI unit that refers to human physiology by incorporating empirical data on spectral sensitivity. V(λ) remains the standard function for imaging and visual display specification in modern technology, ranging from monitors to cameras and smartphones. Another standard observer is the CIE 1931 colourimetric observer [51], describing the spectral sensitivity of human colour vision using standard colour matching functions – $\bar{x}\left(\lambda \right)$, $\bar{y}\left(\lambda \right)$ and$\;\bar{z}\left(\lambda \right)$. The $\bar{y}\left(\lambda \right)$ curve was by design constrained to be the same as V(λ) [51]. It is important to note that these standard observer curves based on data from the UK and the US [52,53] It is clear why standard functions are helpful, namely, to simplify and standardize metrology. At the same time, an average spectral sensitivity curve cannot represent all human responses to light.

The melanopsin spectral sensitivity curve standardized in CIE S026/E:2018 comes closest to these types of standard observers. It shows how a spectrum should be weighted to derive an appropriately weighted quantity. In addition to a standard curve, CIE S026/E:2018 also contains information for generating age-adjusted spectral sensitivity curves, accounting for age-dependent changes lens transmission.

A second and more crucial translational question is how these quantities should be mapped to the physiological response, e.g., the specific effect of light of a given melanopic irradiance. Given the extensive range of individual differences in this response to the same corneal stimulus, a single average dose-response curve will not do the underlying human diversity justice. Instead, we need new ways to make distributional predictions rather than point estimates and communicate these clearly.

Evidence-based recommendations for modifying our light environment and light exposure to enhance its beneficial effects whilst minimizing negative ones are being sought in architectural lighting design, lighting regulations, and building standards [2,54,55]. An international group of experts led by Brown and Wright recently proposed a minimum of 250 lux (melanopic EDI) daytime level, 10 lux evening level and a 1 lux maximum as the night level [56]. These recommendations are tied to a specific observer that is an implied group-average standard model, which makes these criterion light levels appropriate only for this hypothetical observer. Developers of guidelines, regulations, and standards need to be sensitive to such biases in the evidence base.

## 4 The question of participant diversity: ways to move forward

To what extent do data generated in sleep and circadian research reflect the whole range of biological diversity? It is now acknowledged that data on psychological phenomena come from Western, educated, industrialized, rich, and democratic (WEIRD) countries [57], [58], [59], thus creating a biased evidence base. More critically, there is a “sex data gap”, i.e., the underrepresentation of women as participants in biomedical research [60], [61], [62]. Underrepresentation of specific groups of people such as Black, indigenous, and other people of colour has important consequences for biomedical research findings' generalisability [63]. While some studies have addressed differences in circadian physiology across sex (e.g. [64], [65], [66],) and ethnicity (e.g. [67,68],), these are not understood or translated systematically.

Realizing that no single laboratory or research group can fully map out the non-visual responses to light in the face of individual differences and human diversity, a key imperative is to define, explore and evaluate new mechanisms to advance our knowledge. We propose the following steps, which go hand in hand:

• ***Understanding systematic bias in the evidence base:*** When considering the development of guidelines and recommendations for what constitutes "good" light exposure, building upon a demographically biased biomedical evidence base can limit the generalizability and utility of any recommendation. We recommend systematic surveys of the literature to understand the extent to which research on the non-visual effects of light represents diverse populations (for examples of surveys on sex bias in neuroscience and biomedical research, see [62] and the 10-year follow-up [61]). Journal editors or relevant scientific societies could commission such surveys.
• ***Dedicated efforts towards recruiting diverse participants***: To make biomedical research generalizable requires a broad representation of different participants. This could be accomplished through a variety of means [63,69,70], e.g., by appropriate incentives and penalties from funders and institutions, additional research support for developing strategies (e.g., the Wellcome Trust's Diversity & Inclusion scheme, available to Wellcome-funded researchers), patient involvement and co-design mechanisms, and increasing diversity amongst the researchers themselves.
• ***Routine deposition and sharing of data in repositories:*** Data generated in original research should be made available in a well-documented format. This is often described under the heading “open data”. To reflect human diversity in data on the non-visual effects of light requires a data aggregation effort which allows for the combination of data from different participant samples, facilitating novel analyses by participant characteristics that otherwise would not be possible.

Indeed, many journals and funders mandate making data available. Such a mandate can take many forms: Nearly all journals accept supplementary materials, such that data being reported on become part of the report itself. Some publishers also publish data-only journals such as *Scientific Data*. Furthermore, free repositories can be used to store and make available data, including FigShare (https://figshare.org/) and Zenodo (https://zenodo.org/). For sleep data in particular, the NIH-funded *National Sleep Research Resource* (NSRR; https://sleepdata.org/) is a domain-specific example, and NSRR has allowed novel big-data analyses of existing data [71]. We are at present in the early stages of developing a novel *Circadian Data Hub*. There are some concerns regarding the possibility of reidentifying single participants under certain circumstances. These risks need to be carefully navigated, particularly when considering participants who may have experienced discrimination [72].

More generally, and supporting the goal of making data available, the following two action points should also be considered:

• ***Development of meta-data and data standards:*** Sharing of data is only useful when the data are in a format that can be understood. The lack of publicly available or open data in tabulated form indeed may hinder evidence aggregation and synthesis. If data are presented in graphical form (i.e. in a figure in a plot), data points can be estimated using "data thieving" tools like WebPlotDigitizer [73,74].. While this technique can be used to "salvage" published data that have a very low probability of being accessible in tabulated form (for some examples, see [56,^75^,76]), these digital data extraction techniques necessarily lead to information loss. In cases where data points overlap, these techniques are of limited use. Data should be findable, accessible, interoperable, and reusable (FAIR) [77]. One mechanism by which to achieve interoperability is the development of explicit meta-data and data standards. Successful recent examples for this are the Brain Imaging Data Structure (BIDS; [78]) and the Human Cell Atlas meta-data schema (https://github.com/HumanCellAtlas/metadata-schema). Of course, data standards need tooling that makes it easy for data to comply with the standard. Decisions about data organization and storage should be standardized and made accessible. When this is done, it will free up time and resources for other science-related activities.
• ***Improving reporting through the development of domain-specific reporting guidelines:*** Besides storing and organizing data, ways to report and document research also need to be standardized to “future-proof” the data we generate [79]. Guidelines, standard data schemas, and tooling for easy integration into the research workflow will make our science more robust – and more generalizable. Guidelines for reporting light exposure in chronobiology and sleep research have been developed [80,81]. Most recently, the CIE published a technical note on documenting studies examining the non-visual effects of light [82]. Similar recommendations/guidelines exist for actigraphy [83,84] (reviewed in [85]), pupillometry [86] and melatonin measurements [87]. Ideally, guidelines and standards should be developed through a structured process and following recommendations from the EQUATOR (Enhancing the QUAlity and Transparency Of health Research) Network (https://www.equator-network.org/) [88,89].

## 5 Outstanding research questions

In this review, we have highlighted a series of research questions which are key to answer in a principled and systematic way:

• What gives rise to the individual differences in human non-visual sensitivity to light?
• Can these individual differences be related to other factors with genotypic and phenotypic variability?
• How can we build an inclusive evidence base that reflects the entire range of human diversity?
• How do we reflect human diversity and variation in translational applications of research, such as recommendations for lighting and light exposure?

## Conclusion

Research on the non-visual effects of light needs to facilitate the development of an evidence base that recognizes individual differences and variability. We have proposed concrete steps that will help achieve this. Biomedical research on the whole requires wider engagement with diversity and inclusion at all stages of the research life cycle [90], including hiring, funding allocation [91,92], and citation practices [93,94]. Research on the non-visual effects of light is not exempt from that.

## Search strategy and selection criteria

Data for this narrative review were identified by searches of PubMed and Google Scholar, and references from relevant articles using the search terms “non-visual effects of light”, “melanopsin”, “individual differences” and related terms. Searches were also formed based on investigator names. Only full research articles published in English between 1980 and 2021 were included. Articles were chosen according to their relevance to the theme as perceived by the authors.

## Contributors

Conceptualisation: M.S. and N.S.

Funding acquisition: M.S.

Writing – original draft: M.S. and N.S.

Writing – review & editing: M.S. and N.S.

All authors read and approved the final manuscript.

## Declaration of Competing Interest

M.S. is currently an unpaid member of CIE Technical Committee TC 1-98 ("A Roadmap Toward Basing CIE Colorimetry on Cone Fundamentals"). M.S. was an unpaid advisor to the Division Reportership DR 6-45 of Division 3 ("Publication and maintenance of the CIE S026 Toolbox"). Between 2017 and 2020, M.S. was elected Chair of the Color Technical Group within the Optical Society. M.S. is an elected member of the Daylight Academy, an unpaid member of the Board of Advisors of the Center for Environmental Therapeutics, and MS is a member of the Technical Advisory Board of Faurecia IRYStec Inc. Over the past two years (2019-2020), MS has received industrial research support from f.lux software LLC, Ocean Insight, and BIOS Lighting. MS reports funding from the Wellcome Trust, the Royal Society, EPSRC, Bioscientifica Trust, Fight for Sight, Freie Akademische Gesellschaft Basel, and the University of Oxford.

N.S. declares no relevant interests.
